# Rapid Emergence of Chronic Lymphocytic Leukemia During JAK2 Inhibitor Therapy in a Patient With Myelofibrosis

**DOI:** 10.1097/HS9.0000000000000356

**Published:** 2020-05-27

**Authors:** Nikolaos Sousos, Gemma Buck, Alba Rodriguez-Meira, Ruggiero Norfo, Angela Hamblin, Francesco Pezzella, Jennifer Davies, Philip Hublitz, Bethan Psaila, Adam J. Mead

**Affiliations:** 1 MRC Molecular Haematology Unit, MRC Weatherall Institute of Molecular Medicine, University of Oxford, Oxford, UK; 2 Cancer and Haematology Centre, Churchill Hospital, Oxford University Hospitals NHS Foundation Trust, Oxford, UK; 3 Oxford Molecular Diagnostics Centre, Oxford University Hospitals NHS Foundation Trust, Oxford, UK; 4 Cellular Pathology Clinical Service Unit - Haematopathology, John Radcliffe Hospital, Oxford University Hospitals NHS Foundation Trust, Oxford, UK; 5 Haematology Department, Wycombe Hospital, Buckinghamshire Healthcare NHS Trust, High Wycombe, UK; 6 Genome Engineering Facility, MRC Weatherall Institute of Molecular Medicine, University of Oxford, Oxford, UK; 7 National Institute for Health Research Biomedical Research Centre, Oxford, UK

## Abstract

Supplemental Digital Content is available in the text

Myeloproliferative neoplasms (MPN) and lymphoproliferative conditions have been long recognised to co-occur more frequently than might be expected by chance.^1^,^2^ Such patients are not infrequently encountered in clinical practice and present a unique set of diagnostic and management challenges. A particular association appears to occur between chronic lymphocytic leukemia (CLL) and MPN, and such patients have specific clinical and biological characteristics.^2^ A recent study on 1915 patients with MPN showed that those patients have a 2.8-fold greater risk of developing a lymphoid neoplasm compared to the general population,^1^ adding to a series of observations that suggest that MPN and CLL pathobiological processes might be intertwined and their co-occurrence might not be stochastic but rather related to a common pathobiological mechanism promoting MPN and CLL.^1^,^2^ Despite the accumulating evidence of an increased incidence of concomitant MPN and CLL, and some evidence that in many cases the two diseases are genetically distinct,^3^ it remains unclear whether the two disorders arise as subclones from a common ancestral clone or represent 2 genetically independent neoplasms.^2^,^3^

A large retrospective analysis of Philadelphia-negative MPN and lymphoid malignancies reported that the progression and treatment of one disorder does not seem to have an impact on the clinical behaviour of the second disease.^4^ However, with the advent of targeted therapies for both MPN^5^ and CLL,^6^ the potential impact of such therapies in patients with biclonal disease needs careful consideration. In this regard, a recent study has raised the possibility that JAK2 inhibitor therapy might increase the risk of subsequent high-grade transformation of pre-existing low-grade lymphoproliferative conditions.^3^ The potential impact of JAK2 inhibitor therapy in patients with coexisting MPN and CLL remains unknown. Herein we present a patient with post-polycythemia vera myelofibrosis (MF) in whom treatment with the selective JAK2 inhibitor fedratinib resulted in rapid emergence of overt CLL, with an aggressive disease course.

A 72-year-old male was referred to our institute with a diagnosis of previously untreated post-polycythemia vera MF. The polycythemia vera had been diagnosed 22 years previously and was initially managed with regular venesections. Eighteen years after diagnosis the patient developed secondary MF, with anemia, progressive splenomegaly and significant constitutional symptoms. The patient was enrolled in a study of fedratinib, a highly selective JAK2 kinase inhibitor^7^ that has demonstrated spleen and symptom responses in treatment-naïve MF patients^8^ and in MF patients resistant or intolerant to the JAK1/2 inhibitor ruxolitinib.^9^

At the time of commencing trial treatment, physical examination revealed 4 cm hepatomegaly and 19 cm splenomegaly with no lymphadenopathy. Eastern Cooperative Oncology Group (ECOG) score was 0. Full blood count showed white blood cells 8.48 × 10^9^/L, hemoglobin 109 g/L, platelets 114 × 10^9^/L, lymphocytes of 2.37 × 10^9^/L and blood film showed a blast count of 1%. Lactate dehydrogenase was raised at 620 IU/L. Bone marrow trephine was consistent with the diagnosis of overt MF,^10^ with no excess of blasts. Cytogenetics revealed deletion of 20q, which was confirmed by fluorescence in situ hybridization in 41 out of 100 cells examined. The patient was *JAK2V617F* positive with an allelic burden of 75%. Magnetic resonance imaging of the abdomen revealed marked splenomegaly of 24 cm but no lymphadenopathy. The patient was classified as intermediate-2 risk by Dynamic International Prognostic Scoring System (DIPSS)-plus. According to MYSEC-PM (not available at that time) he would be classified as high-risk.^11^

The patient was randomized to 6 cycles of placebo before crossover to fedratinib at 500 mg once a day. At this point, he had a repeat bone marrow evaluation, which remained consistent with the diagnosis of overt MF. The patient had a marked symptomatic improvement on fedratinib treatment, with reduction of spleen size to 12 cm within 2 weeks. Strikingly, in parallel to this response, within 4 weeks of starting fedratinib, the patient's white blood cell count rapidly increased to 16.35 × 10^9^/L with an absolute lymphocytosis of 12.92 × 10^9^/L in comparison with a lymphocyte count of 3.35 × 10^9^/L immediately prior to fedratinib initiation (Fig. 1A-B). Following fedratinib initiation and development of lymphocytosis, blood film evaluation and immunophenotyping established the diagnosis of CLL (see Supplemental Digital Content (SDC) Figure 1, http://links.lww.com/HS/A78, which illustrates CLL immunophenotyping at diagnosis).^12^ Retrospective analysis of the pre-crossover bone marrow trephine biopsy revealed some small foci of lymphoid cell aggregates with CLL phenotype, accounting for approximately 5% of total cellularity with Ki-67 <5% (see Supplemental Digital Content (SDC) Figure 2, http://links.lww.com/HS/A78, which shows CLL pathology findings at diagnosis and pre-treatment initiation).

**Figure 1:**
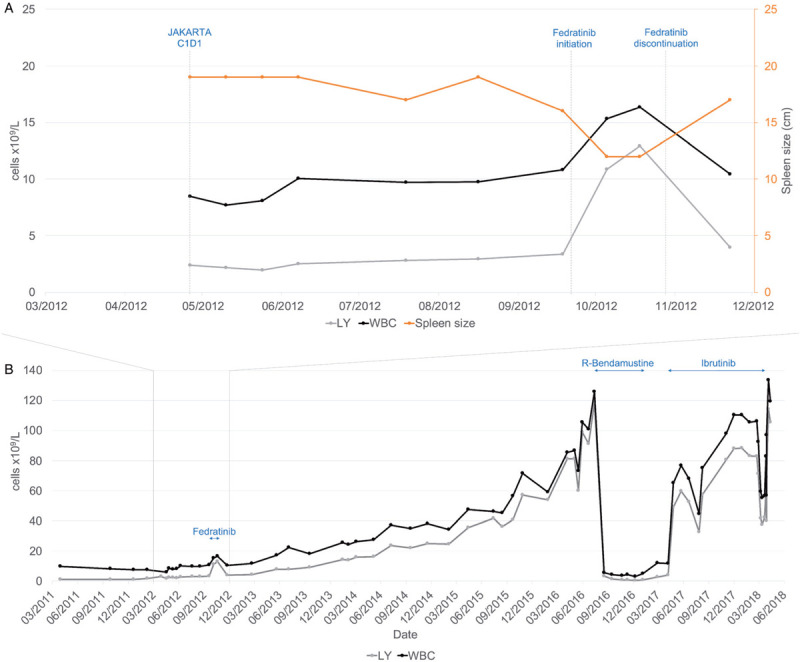
**Timeline of hematopoietic indices.** (A) Differential response of MF and CLL to fedratinib. Line plot shows spleen size by palpation, white blood cell count, and lymphocyte count before, during, and after fedratinib treatment. (B) Longer term follow-up of the above indices. WBC, white blood cells shown in cells ×10^9^/L; LY, lymphocytes shown in cells ×10^9^/L; C1D1, Cycle 1 Day1; R, Rituximab.

The patient was discontinued from the study and promptly stopped fedratinib. Following discontinuation of fedratinib, the patient had a rapid recurrence of splenomegaly and systemic symptoms and in parallel the lymphocytosis was also reduced to 3.97 × 10^9^/L (Fig. 1A-B). Subsequently, the patient was managed with observation rather than immediate treatment for CLL, but developed progressive lymphocytosis (Fig. 1B) and commenced immunochemotherapy with rituximab plus bendamustine due to worsening cytopenias. Pretreatment marrow evaluation showed 100% cellularity with 90% CLL on a background of chronic phase myeloproliferative neoplasm features (see Supplemental Digital Content (SDC) Figure 2, http://links.lww.com/HS/A78, which shows CLL pathology findings at diagnosis and pre-treatment initiation). *TP53* mutational analysis was negative.^12^ The patient completed six cycles of immunochemotherapy and subsequently continued on ibrutinib due to progressing symptomatic nodal disease. After 12 months on ibrutinib the patient had a mixed response but developed severe, refractory headaches. He was diagnosed with subdural hematomas and discontinued ibrutinib for palliation. He died 1 month later.

In order to determine the clonal relationship between the MF and CLL in this patient we carried out additional mutational analysis, including targeted next-generation sequencing for 32 genes commonly mutated in myeloid neoplasms, which did not reveal evidence of additional mutations other than *JAK2V617F*,^13^ and CLL-specific mutational analysis^14^ which identified an *ATM* mutation. The myeloid- and the lymphoid-gene panels were performed in DNA extracted from total nucleated cells in (unsorted) peripheral blood, prior to study enrolment and at CLL diagnosis, respectively. We then purified hematopoietic stem and progenitor cells (HSPC), myeloid cells, T cells and CLL cells (see Supplemental Digital Content (SDC), Materials and Methods, http://links.lww.com/HS/A78). The *ATM* mutation was detected in the CLL compartment but not other cell populations (Fig. 2A). *JAK2V617F* was detected in HSPC and the myeloid cell compartments but not in T-cell and CLL compartments (Fig. 2B-C). Similarly, chromosome 20q deletion was present in HSPC and to a lesser degree in the myeloid compartment but not other populations (Fig. 2D). We conclude that in this patient, the CLL and MF clones were genetically distinct for the driver mutations studied, including the *JAK2V617F* in *JAK2*, the target for fedratinib.

**Figure 2:**
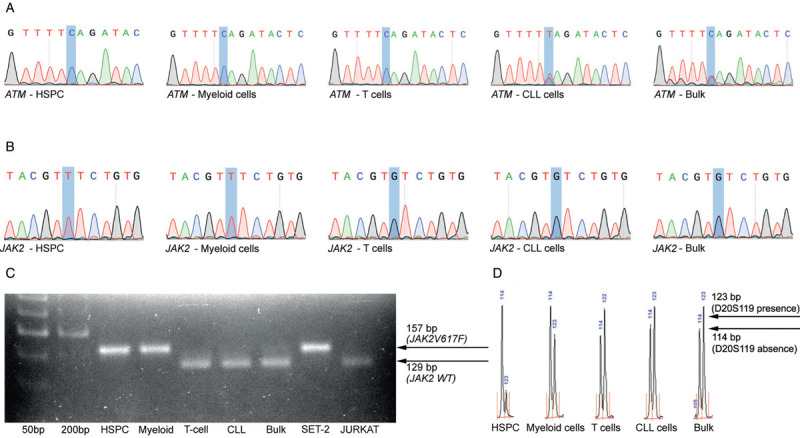
**Evidence of separate clonal origin of MF and CLL.** (A) Sanger sequencing electropherogram, showing *ATM* mutation (NM_000051.3(ATM):c.7181C>T; p.Ser2394Leu) in the CLL compartment but not other indicated cell populations. (B) *JAK2V617F* (NM_004972.3(JAK2):c.1849G>T; p.Val617Phe) was present in high allele burden in HSPC and myeloid compartments but not in T-cell and CLL compartments. (C) Results were confirmed by gel electrophoresis of the AflIII-digested *JAK2* PCR product. The mutant (T) alleles within the HSPC and the myeloid compartments as well as the SET-2 cells remain undigested, yielding a 157 bp fragment. The wild type (G) alleles are cut by the enzyme resulting in a 129 bp fragment, which is the only fragment detected in the T-cell and CLL compartments as well as the JURKAT cells, and the predominant fragment detected in the total mononuclear cells. (D) Capillary electrophoresis for the D20S119 microsatellite in each of the studied cell populations showed the allelic skewing of the 20q region, with a higher percentage of the 114 bp fragment over the 123 bp D20S119 fragment in the HSPC and myeloid compartments, indicating the presence of deletion 20q in those cell populations^3^,^15^.

This case highlights an unexpected and severe adverse response to targeted therapy in a patient with biclonal MF and subclinical CLL. The very clear temporal relationship between JAK2 inhibition and emergence of CLL in this case, including reversibility when JAK2 inhibition was withdrawn, strongly supports a direct link between JAK2 inhibition and CLL progression, although the exact mechanism remains uncertain. This could be caused by a direct effect of JAK inhibition, or possibly an indirect effect due to suppression of the MPN clone. Despite rapid discontinuation of the fedratinib, and early reversibility of the lymphocytosis, the appearance of overt CLL during fedratinib treatment was subsequently followed by an aggressive disease course in contrast to the relevant literature, which suggests that in those cases of concomitant MPN and CLL, the co-occurring CLL typically had a favourable prognosis.^4^ While this cannot be directly attributed to the use of JAK2 inhibitor therapy in this case, it raises a possibility that use of JAK2 inhibitors might lead to irreversible changes in the lymphoid clone, leading to subsequent disease progression. Indeed, it has recently been reported that JAK1/2 inhibition is associated with an increase in the incidence of aggressive B-cell lymphomas, from pre-existing B-cell clones that coexist with MPN.^3^ This report, with fedratinib, a distinct and more selective JAK2 inhibitor,^7^ questions whether this effect is purely related to the immunosuppressive effect of JAK1 inhibitory activity.^3^,^15^ Our case highlights the necessity to carefully evaluate MPN patients for presence of co-existing lymphoproliferative disease, which may be subclinical, and counsel patients in relation to the potential risks of progression of lymphoproliferative disease with JAK2 inhibitor therapy.

## Sources of Funding

This work was funded by a Medical Research Council (MRC) Senior Clinical Fellowship (MR/L006340/1) and a Cancer Research UK (CRUK) Senior Cancer Research Fellowship (C42639/A26988) to AJM, a Hellenic Society of Haematology Foundation Fellowship and a CRUK Oxford Centre Clinical Research Fellowship (C130623/A249471) to NS, and the MRC Molecular Haematology Unit core award (AJM and SEWJ; MC_UU_12009/5). This work was also supported by the WIMM Flow Cytometry Facility, supported by the MRC Human Immunology Unit; MRC Molecular Haematology Unit (MC_UU_12009); NIHR Oxford BRC and John Fell Fund (131/030 and 101/517), the EPA fund (CF182 and CF170) and by the WIMM Strategic Alliance awards G0902418 and MC_UU_12025. The authors would like to acknowledge the contribution of the WIMM Sequencing Facility, supported by the MRC Human Immunology Unit and by the EPA fund (CF268), and they would also like to thank WIMM Single Cell Facility and the MRC-funded Oxford Consortium for Single-cell Biology (MR/M00919X/1) and particularly Dr. Neil Ashley, Facility Manager, for his technical assistance.

## Disclosures

The authors declare the following competing interests: AJM has participated in advisory boards for Novartis, CTI, and Baxaltra; received honoraria from Novartis, Gilead, Shire, and Baxaltra; and also received research funding and travel, accommodation, and expenses from Novartis and Celgene. The other authors declare no conflicts of interest.

## Supplementary Material


