# Correction Notice: Rapid Emergence of Chronic Lymphocytic Leukemia During JAK2 Inhibitor Therapy in a Patient With Myelofibrosis

**DOI:** 10.1097/HS9.0000000000000577

**Published:** 2021-04-21

Since the publication of the article entitled “Rapid Emergence of Chronic Lymphocytic Leukemia During JAK2 Inhibitor Therapy in a Patient With Myelofibrosis” (*HemaSphere.* 2020;4:e356), the authors requested a change to their selected Creative Commons (CC) license. The license previously chosen was CC BY-NC-ND, but is now CC BY to comply with funding mandates. This adjustment has been made and does not affect the outcome of this publication.

The change has been made online: https://journals.lww.com/hemasphere/Fulltext/2020/06000/Rapid_Emergence_of_Chronic_Lymphocytic_Leukemia.9.aspx

