# Supplementary material for: Rapid Emergence of Chronic Lymphocytic Leukemia During JAK2 Inhibitor Therapy in a Patient With Myelofibrosis
Source: Hemasphere. 2020 May 27;4(3):e356. doi: 10.1097/HS9.0000000000000356 (PMC7306308; doi:10.1097/HS9.0000000000000356)
Supplement: Supplementary file 1 [file hs9-4-e356-s001.docx]

**Supplemental Digital Content (SDC).**

**SDC, Additional Clinical Information**

All procedures followed in the present study were performed in accordance with the ethical standards of the current revision of the Declaration of Helsinki. Informed consent was taken for additional genetic analysis carried out (MDSbio study, OXREC C 06/Q1606/110).

The patient’s past medical history included type 2 diabetes mellitus, hypertension, chronic kidney disease stage 3, gout, hypothyroidism, osteopenia, irritable bowel syndrome and hiatus hernia.

At enrollment in the JAKARTA study, concomitant medication consisted of metformin, gliclazide, ramipril, allopurinol, rabeprazole, levothyroxine sodium, adcal-D3, loperamide and mometasone nasal spray. After fedratinib discontinuation the patient did not receive any further treatment for his MF.

At the time of treatment initiation for the CLL, flow cytometry confirmed the CLL clone, accounting for 94% of the lymphoid population while cytogenetics confirmed the presence of del(20q) with no other abnormalities. Treatment consisted of immunochemotherapy with six 28-day cycles of rituximab 500 mg/m^2^ on day 1 and bendamustine 70 mg/m^2^ on days 1,2 of each cycle, followed by 11 30-day cycles of ibrutinib 420 mg once a day (which was complicated with the development of basal cell carcinoma, ophthalmic herpes zoster and subdural hematomas).

**SDC, Materials and Methods**

*Fluorescence activated cell sorting (FACS)*. FACS was performed using the FACSAriaTM III (Becton Dickinson and Company, Franklin Lakes, US-NJ). Cryopreserved peripheral blood mononuclear cells were thawed and processed for flow cytometry analysis as previously described. HSPC were defined as CD3-CD19-CD5-CD23-CD33-CD34+; myeloid cells were defined as CD3-CD19-CD5-CD23-CD34-CD33+; T cells were defined as CD33-CD34-CD19-CD23-CD3+; CLL cells were defined as CD3-CD33-CD34-CD19+CD5+CD23+; bulk consisted of all viable cells. Two hundred cells per population were sorted into phosphate-buffered saline and post-sort FACS purity analysis showed no cross contamination between the HSPC, myeloid, T cell and CLL purified populations.

*Whole genome amplification*. Whole genome amplification for each sorted population was performed using the REPLI-g® Midi Kits (QIAGEN N.V., Venlo, NL) according to the manufacturer's instructions.

*Polymerase chain reaction (PCR)*. Primers for amplification of *JAK2* exon 14 were F; 5’ CAAGCATTTGGTTTTAAATTATGGAGTACGT 3’, R; 5’ TAAATTATAGTTTACACTGACACCTAG 3’ and for ATM exon 49 were F; 5’ AGGCAGTAGAAGTTGCTGGAA 3’, R; 5’ CTGTAGCCCCAGCTATTTCG 3’. To identify allelic skewing in 20q region we used the 20q- microsatellite marker D20S119 with the following primers F; 5’ CTGACACAGTTTCAGTATCTCTATC 3’, R; 5’ TTTCCAGATTTAGGGGTGTATG 3’. Primers were designed to amplify amplicons between 104 and 571 bp and were checked against RefSeq and human genome assembly databases using NCBI Primer-BLAST^®^ (National Library of Medicine, Bethesda, US-MD) as well as SnapGene® Viewer (GSL Biotech LLC, Chicago, US-IL). For restriction digest to identify *JAK2V617F*, the method was as previously described.^1^ All primers were synthesized by Invitrogen^TM^ (Thermo Fisher Scientific, Waltham, US-MA). All PCRs were performed using the KAPA2G Robust HotStart ReadyMix PCR Kit (Merck, Darmstadt, DE) according to the manufacturer's instructions with the following conditions: total volume of 25 μl containing 12.5 μl of the KAPA2G Robust HotStart ReadyMix, 1.25 μl of each primer at 10 μM, template DNA as required and PCR-grade water up to 25 μl; and the following cycling protocol: initial denaturation for 3 minutes at 95^o^C, 35 cycles of denaturation for 15 seconds at 95^o^C, annealing for 15 seconds at 60^o^C and extension for 15 seconds at 72^o^C, and final extension for 1 minute at 72^o^C. All PCR amplicons were purified using Agencourt® AMPure® XP Magnetic Beads (Beckman Coulter, Inc, Brea, US-CA) according to the manufacturer’s instructions.

*Gel electrophoresis.* Initial assessment of DNA fragment size was performed using gel electrophoresis on a 3% ethidium bromide-containing (10^-4^ g/L) agarose gel in TAE buffer (Tris Acetate-EDTA, Tris acetate 40 mM, 1mM EDTA, pH 8.3) at 120 mV for 30 minutes. Approximate DNA fragment size was determined using a 50 bp ladder (PCRBIO Ladder III, PCR Biosystems Ltd, London, UK).

*Sanger DNA Sequencing*. Sanger DNA sequencing was applied for the detection of *JAK2* c.1849G>T p.V617F and the *ATM* mutations in each of the different cell compartments. Sequencing was performed by the Applied Biosystems^TM^ 3730 DNA Analyzer (Thermo Fisher Scientific, Waltham, US-MA) with use of the BigDye® Terminator v3.1 chemistry. Results were visualized and annotated using the SnapGene® Viewer software (GSL Biotech LLC, Chicago, US-IL).

*Capillary electrophoresis*. Separation, identification and quantitation of both D20S PCR product fragments and restriction enzyme digest products were performed with use of the automated fluorescence-based capillary electrophoresis system Fragment Analyzer^TM^ (Advanced Analytical Technologies, Inc., Ankeny, US-IA) and the dsDNA 905 reagent kit of 35 bp – 500 bp sizing range.

**References**

1. Lambert JR, Everington T, Linch DC, Gale RE. In essential thrombocythemia, multiple JAK2-V617F clones are present in most mutant-positive patients: a new disease paradigm. *Blood* 2009;114(14): 3018–3023.

**SDC, Figures**

**
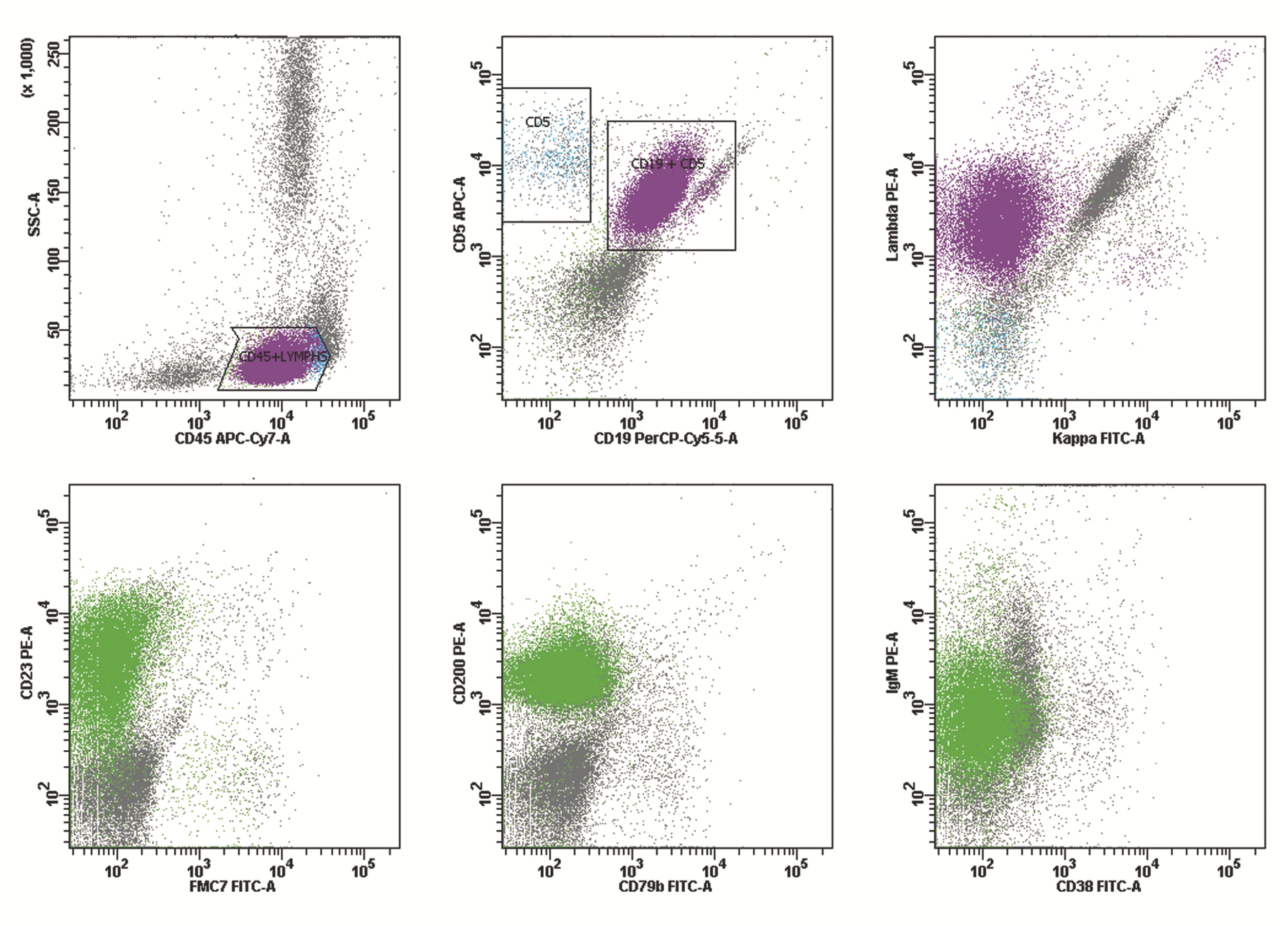
**

**SDC, Figure 1. CLL immunophenotyping at diagnosis.** Immunophenotyping at diagnosis of CLL showing a clonal B-cell population, positive for CD19, CD5, Lambda, CD23, CD20, CD200, weak sIgM, negative for FMC7, CD79b, CD10, CD38, approximately 60% of lymphoid gated events.

**
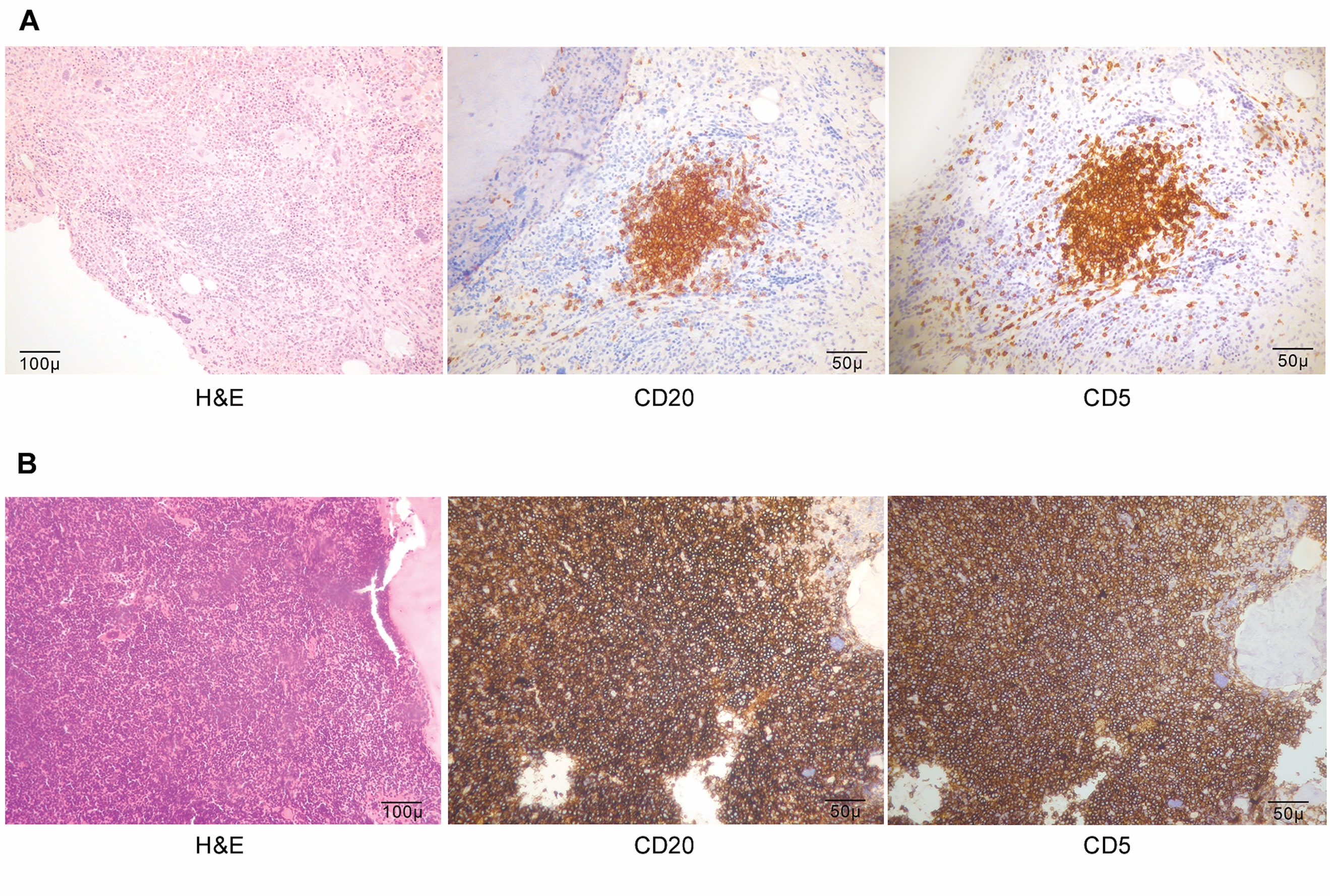
**

**SDC, Figure 2. CLL pathology findings at diagnosis and pre-treatment initiation.** (A) Retrospective analysis of the pre-study bone marrow trephine revealed focal CLL aggregates (positive for CD20, CD79a, CD5, CD23, negative for CD3, CD10, cyclin D1), accounting for approximately 5% of total cellularity. (B) Bone marrow evaluation 42 months later, at CLL treatment initiation, showed approximately 95% CLL marrow infiltration.
